# Urinary MicroRNA Sensing Using Electrochemical Biosensor to Evaluate Colorectal Cancer Progression

**DOI:** 10.3390/biomedicines10061434

**Published:** 2022-06-17

**Authors:** Sow-Neng Pang, Yu-Lun Lin, Yueh-Er Chiou, Wai-Hung Leung, Wen-Hui Weng

**Affiliations:** 1 Department of General Medicine, Mater Misericordiae University Hospital, D07 R2WY Dublin, Ireland; benpang2004@gmail.com; 2 Department of Chemical Engineering and Biotechnology and Graduate Institute of Biochemical and Biomedical Engineering, National Taipei University of Technology, Taipei City 106, Taiwan; 11xc11860324@gmail.com; 3 Department of Nursing, College of Medicine, Fu Jen Catholic University, New Taipei City 242, Taiwan; 031051@mail.fju.edu.tw; 4 Division of Colorectal Surgery, Department of Surgery, Mackay Memorial Hospital, Taipei City 104, Taiwan

**Keywords:** biosensor, miR-21, miR-141, colorectal cancer, nucleic-acid sensor

## Abstract

Research in cancer diagnostics has recently established its footing and significance in the biosensor sphere, emphasizing the idea of a unique probe design used as a sensor and actuator, to identify the presence of protein, DNA, RNA, or miRNA. The fluorescein isothiocyanate (FITC) probe and biotinylated probe are designed for a two-pronged approach to the detection of the urinary miR-21 and miR-141, both of which have demonstrated significance in the development and progression of colorectal cancer, a leading cause of mortality and morbidity. The remainder of the apparatus is composed of a modified screen-printed carbon electrode (SPCE), to which the probes adhere, that transduces signals via the redox reaction between H_2_O_2_ and HRP, measured with chronoamperometry and cyclic voltammetry. The precise nature of our ultra-non-invasive biosensor makes for a highly sensitive and practical cancer detector, concluded by the significance when establishing disease presence (miR-21 *p*-value = 0.0176, miR-141 *p*-value = 0.0032), disease follow-up (miR-21 *p*-value = 0.00154, miR141 *p*-value < 0.0005), and even disease severity. This article hopes to emphasize the potential of an additional clinical tool for the management of colorectal cancer.

## 1. Introduction

According to statistics from the Global Cancer Observatory in 2020, colorectal cancer (CRC) ranks as the third commonest cancer in the world [1], and second in cancer-related mortality rate. Today’s CRC screening methods can be divided into two categories: invasive and non-invasive testing. Non-invasive tests include stool and blood tests and radiological imaging. At present, the commonly used stool tests are mainly the guaiac-based fecal occult blood test (gFOBT) [2] and fecal immunochemical test (FIT) [3]. The gFOBT uses the chemical reaction of peroxidase activity in heme to detect the presence of blood in the stool. It is a cheap, simple and widely available test. The concept is to extract blood samples and cell debris related to polyps and cancer [4]. Radiological examinations include capsule endoscopy and computed tomographic colonography (CTC) [5]. These are used to observe the changes in the intestinal mucosa and to suggest the presence of cancer through radiographic imaging. Invasive examinations include flexible sigmoidoscopy (FS) and colonoscopy [6]. In recent years, many countries have adopted this method as the main screening tool. This procedure allows for the direct visualization and sampling of pathological sites and is the main diagnostic procedure for colonic polyps and advanced tumors.

Compared with other potentially harmful or expensive screening methods, urine has the advantages of non-invasiveness, low cost and ease of access [7]. At present, the main examination of residual urinary-tract epithelial cells, urine protein and other urine metabolites in urine such as tyrosine, citrate, tryptophan, valine, and other organ-malignant tumor indicators are used as a way to diagnose cancer [8,9]. In cancers related to the urinary-tract system, such as bladder cancer, kidney cancer and prostate cancer, the tumor DNA can be extracted from the urine, and using its concentration, mutations and methylation status, stages of cancer can be assessed. It is currently used in clinical practice for bladder cancer [10,11]. Urinary protein screening is also standard practice in monitoring and screening for kidney cancers [12]. In recent years, several teams have studied microRNA in urine exosomes and have concluded its association with breast cancer [13], ovarian cancer [14], and pancreatic cancer [15], all showing significant correlations.

Currently, common nucleic-acid-detection methods are fluorescent in situ hybridization, microarray, reverse transcription PCR, or next-generation sequencing, amongst other diagnostic techniques [16,17]. However, most of these techniques require careful pre-processing of the sample and are therefore generally not suitable for immediate analysis. In recent years, there has been tremendous interest and development in biosensors focusing on nucleic-acid detection, such as in cancer research [18], SARS-CoV-2 detection [19], and even environmental pollution monitoring [20], etc., due to their cost efficiency, convenience, portability, low complexity, and their use of signal amplification to provide rapid detection with higher sensitivities. Commonly used nucleic-acid biosensors are composed of nucleic-acid-recognition elements and signal transduction. The principle is to use single-stranded DNA (ssDNA) molecules as probes [21]. The nucleic-acid sequence of the probe will hybridize with the complementary target nucleic-acid sequence to form specific hydrogen bonds. Upon hybridization on the surface of the transduction material, the enhanced signal will be converted into a current response by electrochemistry [22,23,24]. Several signal-amplification methods for nucleic-acid detection have been reported, such as enzymes and other nanomaterials. The amplified signals were quantified using electrochemical analysis techniques, such as chronoamperometry (CA), differential pulse voltammetry (DPV), cyclic voltammetry (CV) and electrochemical impedance spectroscopy (EIS) [25].

MicroRNA is a small non-coding regulatory RNA molecule whose main function is to regulate the post-translational modification of genes. Its function can effectively lead to the degradation of target messenger ribonucleic acid (mRNA), thereby inhibiting the performance of target genes [26]. Not only is it involved in the process of regulating cell proliferation, differentiation and apoptosis, but it may also provide a similar role to tumor-suppressor genes and oncogenes [27]. The expressional type of miRNA can correlate to several cancers, stages of cancers, and other clinical variables. The analysis of miRNA expression also shows the carcinogenic or tumor-suppressor effect of miRNA [28,29]. Since miRNA analysis can be used as a tool for cancer diagnosis and prognosis, the means to develop a fast and sensitive measurement method is now a major challenge [30]. miRNA is commonly present in tissues and bodily fluids, such as plasma, serum and urine [28,31]. Urine contains a large amount of nuclease, but it does not affect the expression of miRNA in urine [32,33]. miRNA is secreted into the circulation through many different mechanisms [34]. Coupled with this, the degradation of miRNA through endogenous RNase has proven to be resistant and difficult due to the length of the nucleic acid, its encapsulation through exosome microvesicles, and its existence in very stable forms in bodily fluids, including serum and urine [35]; therefore, it is now believed that the study of miRNA content in bodily fluids can be used as a major biological indicator for various diseases, and its association to cancer is undeniable [36,37,38], including colorectal cancer [39], gastric cancer [40], prostate cancer [41], breast cancer [42] and ovarian cancer [43].

The presence of miRNA in bodily fluids and its association to cancer is undeniable. In a previous experiment, our laboratory successfully developed a biosensor for miR-141, which is predominantly expressed in breast cancer [44] and prostate cancer [45], with comparatively less expression in colorectal cancer in serum [46]. Therefore, prompting this experiment, a nucleic-acid sensor was designed for miR-21, as its involvement in colorectal cancer has been well established from disease oncogenesis, proliferation and invasion, to treatment resistance [47,48].

## 2. Materials and Methods

### 2.1. Clinical Urine-Sample Collection

In total, 10 colorectal cancer patients and 10 normal people (confirmed without CRC, then used as control) were involved in the current study. The timing of urine collection from CRC patients was once before operation, and post-operation urine collections at day 3, day 7 and one month. All were random specimens that were collected when patients came to the clinic. This study was carried out with the approval of the Human Subject Research Ethics Committee/Institutional Review Board (IRB: 20MMHIS201e) of Mackay Memorial Hospital (Taipei, Taiwan).

### 2.2. Design of Oligonucleotides as Biomarkers and Probes

With the screen-printed electrode as the established transduction platform, the newly designed miR-21 and miR-141 sensor is a two-pronged electrochemical nucleic-acid sensor. Two complementary sequences to miR-21 and miR-141 were designed and identified as a detector probe (Fluorescein -5′-TCAACATCAGT-3′, and Fluorescein -5′-CCATCTTTAC-3′) and a capture probe with biotin modifications (CTGATAAGCTA-3′-biotin, and 5′-CAGACAGTGTTA-3′-biotin); with the capture probe fixed on the electrode surface to provide current signals once hybridization occurs.

### 2.3. Screen-Printed Carbon Electrode Modification with ssDNA Probe

The water used in this experiment was treated with DEPC (use concentration was 0.1% *v*/*v*) to remove RNA-degrading enzymes in the water. The surface of the carbon electrode was modified by adding DEPC, autoclaved and shaken overnight, then instilled with 30 μL of 50 mg/mL carboxymethyldextran sodium salt (CMD) on the surface and left to react for 16 h. CMD forms COOH functional groups on the surface of the carbon electrode and activates the surface of the electrode, which facilitates subsequent chemical modifications. The residual CMD solution was removed with PBS washes, repeated 3 times. A preparation of EDC-NHS solution was configured with 8 mg/mL of EDC and 22 mg/mL of NHS mixed with 0.1 M pH 4.7 MES buffer. Next, 30 μL of this solution was instilled on the surface of the carbon electrode and reacted at room temperature for 15 min. EDC-NHS will react with the COOH functional groups on the surface of the carbon electrode to produce a stable chemical structure of acyl groups on the surface. Residual EDC-NHS solution was removed with PBS wash as above. Next, 30 μL of 10 μL/mL Neutravidin solution was instilled on the surface of the carbon electrode and left to react at room temperature for 1 h. Neutravidin will replace the surface base, which will facilitate subsequent biotinylated ssDNA probe binding. The electrode was washed 3 times with PBS to remove residual Neutravidin, and 20 μL of 5 μM biotinylated ssDNA probe was instilled on the surface of the carbon electrode and left to react at room temperature for 1 h. The biotinylated ssDNA probe will react with the Neutravidin binding site to ensure firm attachment to the electrode surface. The unbound biotinylated ssDNA probe was removed with 3 washes with PBS. Next, 20 μL of 1 M Ethanolamine solution was dropped onto the surface of the carbon electrode, for a reaction time of 10 min at room temperature. Ethanolamine acts to block non-specific junction areas. The electrode was once more washed with PBS 3 times to remove unbound Ethanolamine, and was then able to be used as a miRNA-detection platform.

### 2.4. Completely Modified Screen-Printed Electrodes to Capture Target miRNA

The 1 µM FITC probe and specimen were hybridized in STE buffer (0.1 M NaCl, 10 mM Tris, 1 mM EDTA, pH 8.0), heated at 55 °C for 3 min, and left to react at 4 °C in the dark for 15 min. Next, 20 µL of the sample was injected into the working electrode and left for 15 min. One round of PBS wash was required to remove the unbound specimen, and then 20 μL of 1 μg/mL anti-fluorescein HRP solution was applied to the electrode and kept in the dark for 15 min, followed by a final PBS wash prior to electrochemical detection.

### 2.5. miRNA RT-qPCR

According to TaqMan^®^ microRNA Reverse Transcription Kit (Thermo Scientific™ 4366596, Waltham, MA, USA), the recommended amount of total RNA is 10 ng/15 μL. There are two sets of TaqMan™ MicroRNA Assays, hsa-miR-21 (Thermo Scientific™ 000397, Waltham, MA, USA) and hsa-miR-141 (Thermo Scientific™ 000463, Waltham, MA, USA). In our experiment, we took 2 μL of urine sample and placed it in a U-shaped bottom 96-well dish, added 48 μL of nuclease-free water, and placed it in an ultra-micro spectrophotometer to measure out the RNA concentration at 260 nm. To obtain the total RNA concentration of the stock solution, we subtracted the background value by the OD260 absorbance value and multiplied the conversion factor (40 μg/mL for single-stranded DNA to RNA) and the dilution ratio. Next, 10 ng of RNA was mixed with nuclease-free water to make 5 μL. In order to improve the binding rates of the RT primer and miRNA, we first added 3 μL of 5 RT primer to bind with RNA, and left it to react at 85 °C for 5 min, 60 °C for 5 min, and finally it was reduced to 4 °C. The miRNA reverse transcription reagents were configured as such: TaqMan™ Fast Advanced Master Mix, 10 μL per well; nuclease-free water, 7 μL per well; TaqMan Small RNA assay, 20 × 1 μL per well; RT-Product, 2 μL per well. Then, 7 μL of reverse-transcription reagents were added to each tube. Next, it was placed into the PCR machine (Applied Biosystems Veriti Thermal Cycler) at 16 °C for 30 min, 42 °C for 30 min, 85 °C for 5 min, and finally reduced to 4 °C. After transcription, the cDNA can be stored in the refrigerator at −20 °C.

### 2.6. Analysis Device and Software

This study used Metrohm Autolab PGSTAT204 for electrochemical detection. The software for operation and analysis was NOVA1.11 (Utrecht, The Netherlands). The electrochemical experimental methods used were cyclic voltammetry (CV), linear sweep voltammetry (LSV) and chronoamperometry (CA). The data-sorting software was Origin9.0. The electrochemical detection was carried out in the electric box, which reduces the environmental factors. This experiment was analyzed by the three-electrode method. The working electrode was a screen electrode, the auxiliary electrode was a platinum wire, and the reference electrode was a calomel electrode. This experiment used two types of electrolytes: 5 mM Ferricyanide dissolved in 0.1 M KCl, and TMB/H_2_O_2_ solution.

### 2.7. Statistical Methods

In regard to electrochemical measurements from the sensor used, the cyclic voltammetry method selects the current-density value under the 4th loop reduction potential, the linear sweep method uses the highest peak value, and the chronoamperometry method uses the 200 s terminal current value. A triple repeat of the experiment was used to calculate the standard deviation. RT-qPCR sets the number of replication cycles (ct value) measured by healthy subjects to 1, subtracts the number of replication cycles measured by other subjects and calculates the exponential subtraction loop of 2 to push back the relative gene expression. GraphPad Prism 9.0 software (San Diego, CA, USA) was used to make interquartile range and scatter plots, and the *p*-value was calculated by using the t-test and ANOVA single factor.

## 3. Results

### 3.1. Triple Repeat Experiment for Mean and Standard Deviation

**Figure 1:**
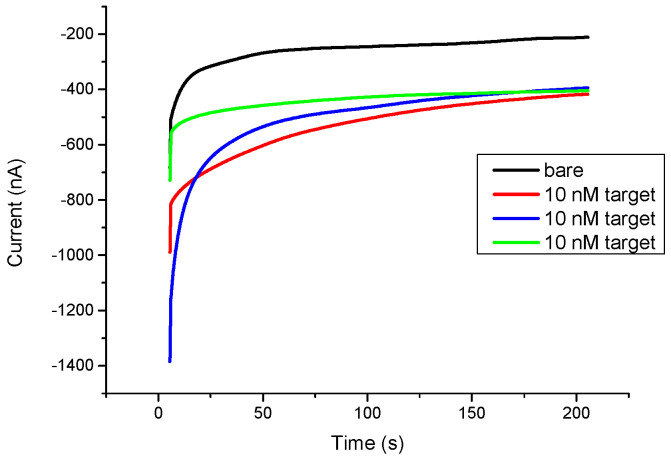
A stability triple repeat test of the miR-21 biosensor against 10 nM of miR-21, recorded with chronoamperometry.

The screen-printed electrode with a modified biotin probe binds to the target miR-21 that has been bound to the fluorescein probe and reacts in an electrolyte mixture with 0.4 mM horseradish peroxide and 0.4 mM hydrogen peroxide. After applying a voltage of −0.3 V and a reaction of 200 s, using chronoamperometry, the miR-21 biosensor was able to output a response of 400 nA after target capturing, with approximately 350 nA terminal current stability, compared to the control with only surface functional modification and no target annealing, which outputted 200 nA and a 250 nA terminal current stability (Figure 1).

**Figure 2:**
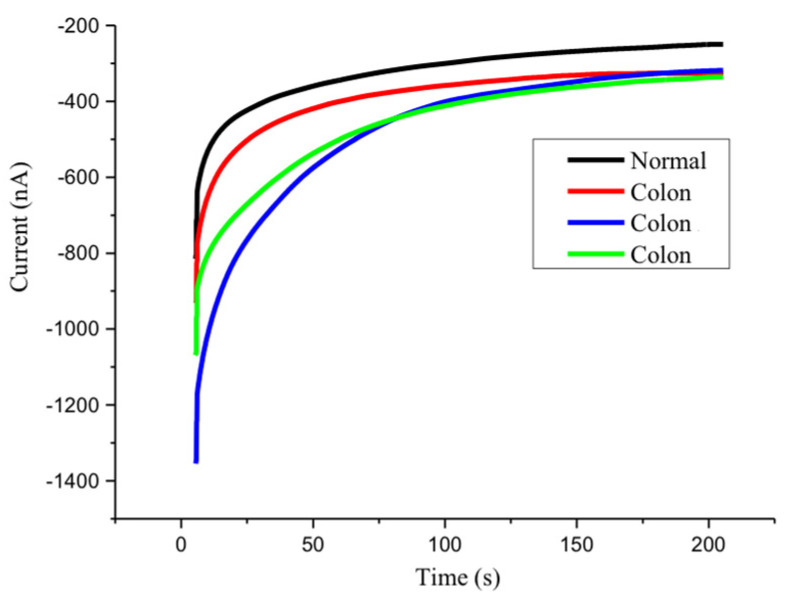
Stability triple testing of the miR-21 biosensor using a single colorectal cancer patient and normal urine sample, recorded with chronoamperometry.

The fully modified miR-21 sensor was used on colorectal cancer patients’ and healthy subjects’ urine samples for chronoamperometric measurement. It was found that colorectal cancer urine samples can obtain approximately 350 nA terminal stability after each test, while the healthy urine samples only measured 250 nA terminal stability (Figure 2). It is clear that the newly developed sensor can reliably capture miR-21 in urine, whilst also displaying characteristics of high expression of miR-21 in colorectal cancer patients’ urine.

### 3.2. Selectivity

**Figure 3:**
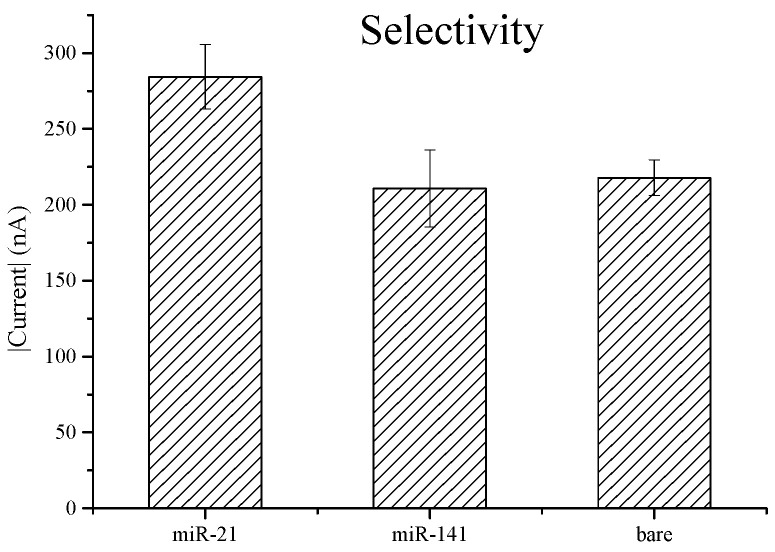
miR-21 nucleic-acid sensor selectivity comparison in 10 mM of miR-21, 10 mM of miR-141, and bare electrode using chronoamperometry.

The modified electrode was tested against a 10 nM miR-21 sample, 10 nM of miR-141, and a control electrode with no miRNA; the results were analyzed by chronoamperometry. From the stable current values, as expected, there was a higher current value from the miR-21 electrode than from the latter two electrodes, approximately 300 nA and 200 nA, respectively, with no overlapping current values between the subject miRNA and comparison subjects in repeat testing (Figure 3). This confirms a highly selective biological sensing device.

### 3.3. Sensitivity

**Figure 4:**
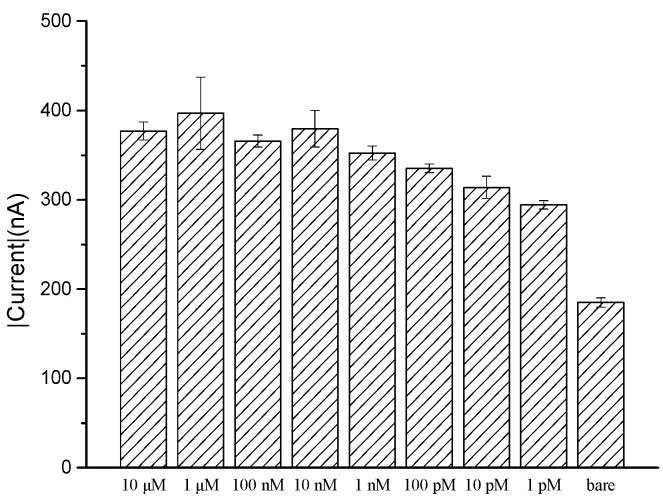
Corresponding current levels to miR-21 concentration using chronoamperometry.

A range of concentrations of miR-21 were tested using the modified screen-printed electrode to determine sensitivity and the lowest reliably detectable concentration; the current response was analyzed by chronoamperometry. It was observed that in a high concentration range of 10 μM to 10 nM, miR-21 can measure nearly 400 nA, with overlapping results between repeat tests. A ten-fold incremental reduction in concentration was assessed to the lowest 1 pM. It was found that there was a concurrent downward trend in the low concentration range of 10 nM to 1 pM. However, at the lowest 1 pM concentration, it still produced a 300 nA current, which was higher than our bare electrode and higher than the previously mentioned 250 nA stable current observed in our selectivity experiment (Figure 4). Therefore, the concentration detection limit proposed through this experiment has a lower limit of 1 pM and an upper limit of 10 nM to reach saturation.

### 3.4. SPCE Versus the Gold Standard RT-qPCR in miRNA Detection

**Figure 5:**
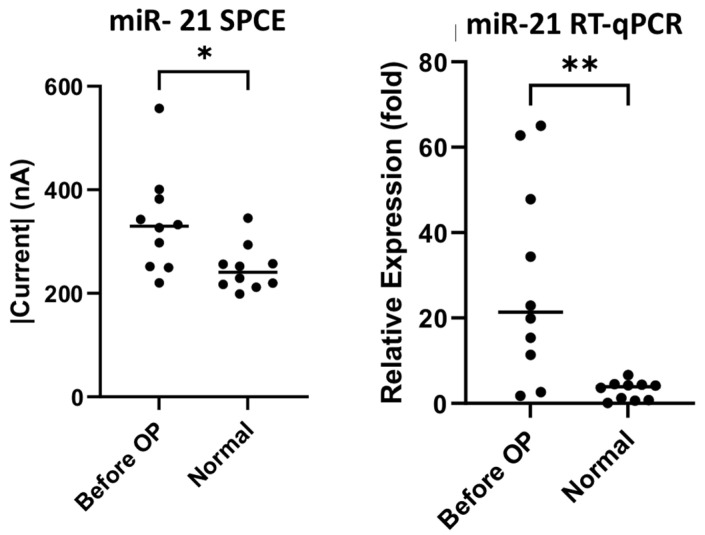
miR-21 sensor detection and RT-qPCR analysis of urine samples of colorectal cancer patients and healthy subjects. * *p*-value < 0.05, ** *p*-value < 0.005.

**Figure 6:**
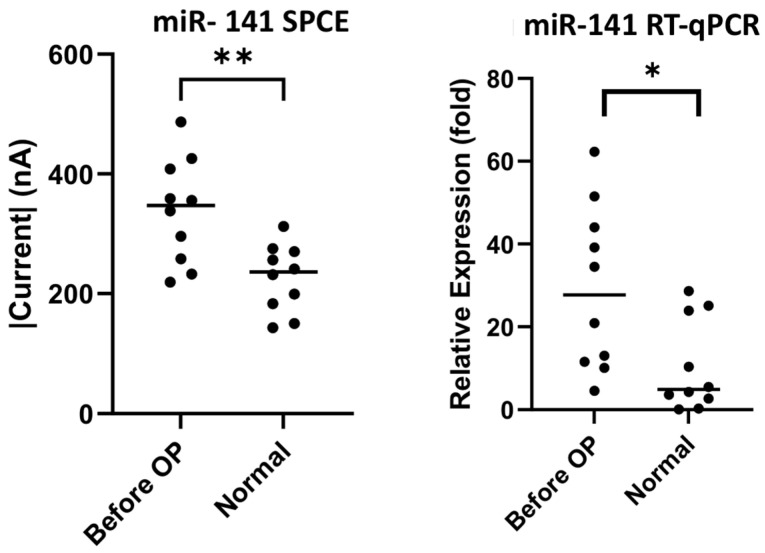
miR-141 sensor detection and RT-qPCR analysis of urine samples of colorectal cancer patients and healthy subjects. * *p*-value < 0.05, ** *p*-value < 0.005.

Ten samples each of preoperative colorectal cancer patients’ and healthy subjects’ urine were collected for preliminary comparison using both the biosensor and RT-qPCR. In the detection of miR-21 there were significant differences, as seen in Figure 5 (SPCE *p*-value = 0.0176, RT-qPCR *p*-value = 0.003), as well as in the detection of miR-141, as seen in Figure 6 (SPCE *p*-value = 0.0032, RT-qPCR *p*-value = 0.0180). The result confirms that our nucleic-acid sensor can reliably distinguish the difference between cancer patients and healthy subjects.

### 3.5. miRNA Sensor in the Follow-Up of Colorectal Patients

**Figure 7:**
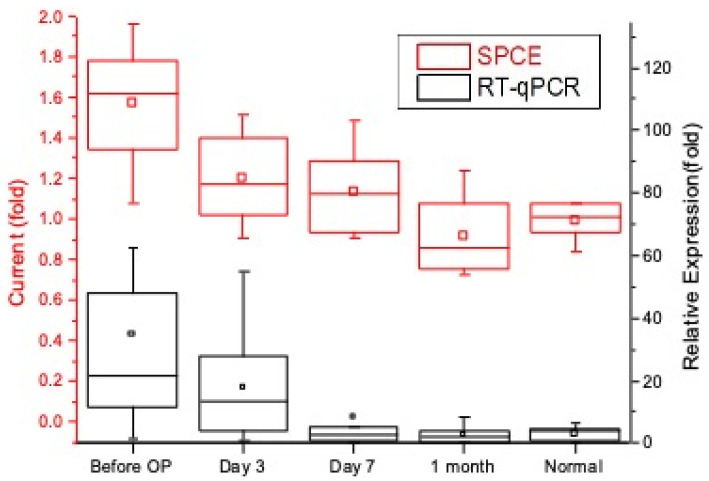
Detected miR-21 in colorectal cancer patients before and after surgery with RT-qPCR analysis.

**Figure 8:**
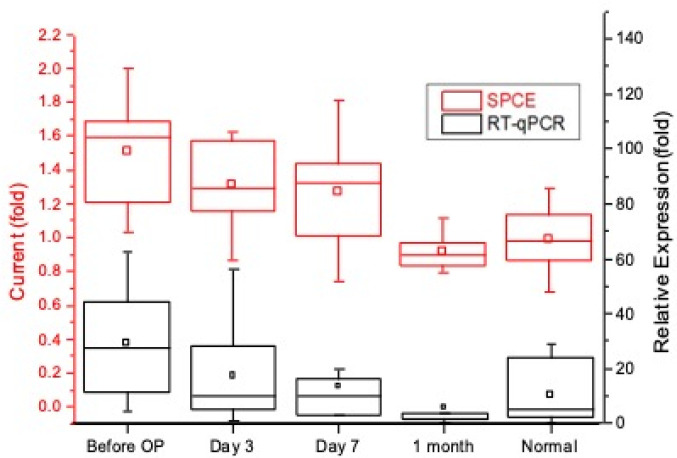
Detected miR-141 in colorectal cancer patients before and after surgery with RT-qPCR analysis.

This experimental study uses nucleic-acid sensors to perform pre- and postoperative short-term analysis of urinary miR-21 and miR-141 between 10 groups of colorectal cancer patients and healthy subjects. RT-qPCR was used for comparison, and healthy subjects were set as the reference value of 1. In both the miR-21 and miR-141 experiments, it was found that the terminal stability current of the 10 groups of patients had a higher trend before their operation compared to the postoperative phases. This is concurrent with the qPCR results. Within the one-week postoperative phase, it seems that there was no significant reduction in target nucleic acids on day seven (miR-21 *p*-value = 0.102, miR-141 *p*-value = 0.267); however, there were significantly steady low levels after one month into the postoperative phase, especially when looking at miR-141 (miR-21 *p*-value = 0.00154, miR-141 *p*-value = 0.00001); the levels were almost comparable to those of the healthy subjects (Figure 7 and Figure 8). From this experiment, it is evident that our biosensor is able to detect an increase in target nucleic acids, which establishes its usefulness in the pre- and postoperative evaluation of colorectal cancer patients.

### 3.6. miRNA Levels Compared to Cancer Stages

**Figure 9:**
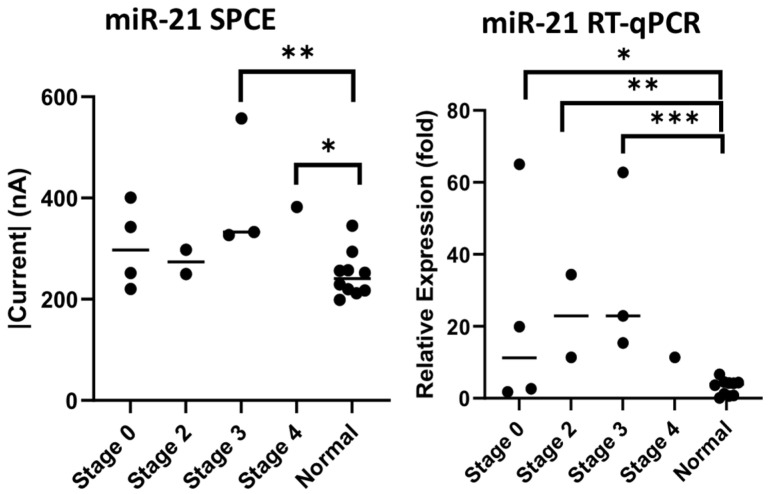
Measurement of miR-21 by cancer staging through miR-21 SPCE and RT-qPCR. * *p*-value < 0.05, ** *p*-value < 0.005, *** *p*-value < 0.0001.

**Figure 10:**
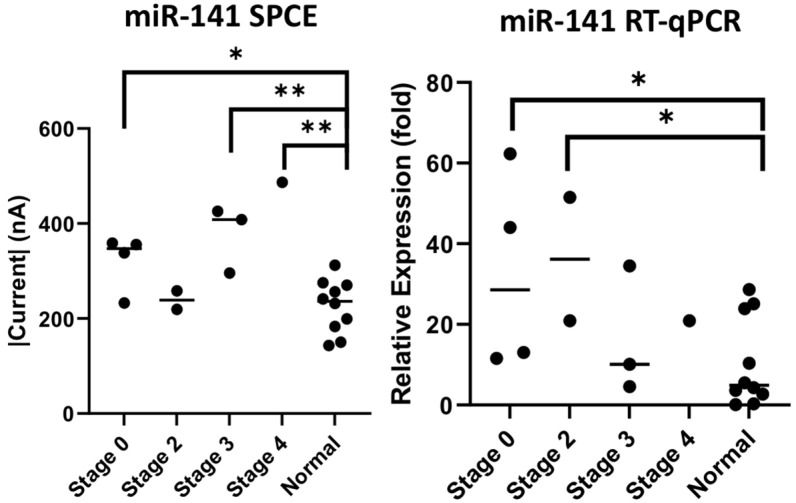
Measurement of miR-141 by cancer staging through miR-141 SPCE and RT-qPCR. * *p*-value < 0.05, ** *p*-value < 0.005.

Cancer stages and their associations with levels of mRNA were also investigated in this experiment. The urine samples of the ten patients with colorectal cancer collected in this study included four patients at stage 0, two patients at stage 2, three patients at stage 3, and one patient at stage 4. Compared to cancer-free urine samples, we observed significant levels of expression using both our nucleic-acid sensor and RT-qPCR. Through our biosensor, patients in stages 3 and 4 measured higher current values with significance, as seen in Figure 9 (stage 3 *p* = 0.0052, stage 4 *p* = 0.0176), and from RT-qPCR, there were also significantly higher levels of expression of miR-21 in stages 0, 2, and 3 (stage 0 *p* = 0.05, stage 2 *p* = 0.001, stage 3 *p* = 0.0014). Similarly, in the miR-141 results (Figure 10), through the biosensor we found higher current values detected in the zero, third and fourth stages of colorectal cancer (stage 0 *p* = 0.0152, stage 3 *p* = 0.0026, stage 4 *p* = 0.0016), and RT-qPCR also showed higher expression of miR-141 in the zero and second stages (stage 0 *p* = 0.0331, stage 2 *p* = 0.0244).

The mean and standard deviation of each cohort was calculated to assess result variability in relation to cancer stages. The data were calculated using fold change, with the mean of normal samples as the denominator. Unfortunately, there was only one data point for stage 4, therefore we omitted the data from this study. From the miR-21 results, we observed a low mean and standard deviation on our biosensor (stage 0 (1.22 ± 0.288), stage 2 (1.10 ± 0.096), stage 3 (1.63 ± 0.432)) compared to the RT-qPCR results (stage 0 (25.7 ± 22.3), stage 2 (22.9 ± 11.5), stage 3 (33.7 ± 20.8)). This outcome was similarly seen in the miR-141 result, with our biosensor producing lower means and standard deviations (stage 0 (1.42 ± 0.229), stage 2 (1.05 ± 0.086), stage 3 (1.66 ± 0.255)) compared to RT-qPCR (stage 0 (32.7 ± 21.4), stage 2 (36.2 ± 15.3), stage 3 (16.4 ± 13.0)) (Figure 9 and Figure 10).

## 4. Discussion

The primary objective of this study was to establish the novelty of this urinary miR-21 nucleic-acid biosensor, and its potential for clinical application. We can definitely see significant results for miR-21 detection through this study. Our laboratory decided to probe further, and we attempted to establish cancer staging and its relation to the presence of mRNA. The significance of mRNA presence in colorectal cancer from a pathological, staging and post-tumor-resection perspective was previously documented by a research team who conducted RT-qPCR analysis on serum samples of colorectal cancer patients, showing high correlations and significant down-regulation of seven mRNAs including our subject, miR-21 [49]. Understanding the properties of miR-21 and discerning its concentration in urine through our biosensor to compare with RT-qPCR, as seen in Figure 5, showed significant differences between normal specimens and CRC specimens (SPCE *p*-value = 0.0176, RT-qPCR *p*-value = 0.003), as did the detection of miR-141, as seen in Figure 6 (SPCE *p*-value = 0.0032, RT-qPCR *p*-value = 0.0180); in addition, Figure 9 and Figure 10 reinforce the significance that this research and development has on the future of colorectal cancer staging using the urinary detection of miR-21 and miR-141 (miR-21 stage 3 *p* = 0.0052, stage 4 *p* = 0.0176, and miR-141 stage 0 *p* = 0.0152, stage 3 *p* = 0.0026, stage 4 *p* = 0.0016). Interestingly, when comparing results from our biosensor to RT-qPCR, the data variability, defined as mean and standard deviation, was wider in the RT-qPCR results in both the miR-21 and miR-141 cohort when compared to our nucleic-acid biosensors (Figure 9 and Figure 10). Knowing that miR-21 levels are highly expressed in advanced diseases in many forms of cancers including colorectal cancer, and its concentration correlates to tumor progression and regression [50], we propose that the developed biosensor has potential to be a more accurate predictor of colorectal cancer staging than that of the traditional PCR. To clarify its significance, further studies with larger sample sizes are necessary to establish finer margins of current thresholds from urinary samples in order to accurately correspond to cancer stages; furthermore, it is proposed from this research that a combined miRNA biosensor of miR-21 and miR-141 on a singular modified SPCE might accentuate the margins and provide more accurate results to identify cancer in its stages.

The range of clinical applications for the new biosensor have proven to be an imposing competitor to our current methods of first-line screening of colorectal cancer: the fecal immunochemical test (FIT) and the fecal occult blood test (FOBT). It is evident that there are high levels of false negatives from these tests, especially in cancers that do not bleed or in patients that have other risk factors, i.e., smoking, hyperglycemia, and hypertension, etc. [51,52]. It is our hope that with further high-quality, high-volume studies, hopefully in conjunction with the current methods for screening, the proposed biosensor will have a place in the future of colorectal cancer screening, diagnosis, and follow-up. At the moment, although promising, we can only confidently establish the presence of colorectal cancer in the binary sense.

## 5. Conclusions

This work provides a fundamental for the future development of nucleic-acid-based biosensors for the primary purposes of cancer screening, diagnosis, staging and follow-up. The newly developed two-pronged biosensor by our laboratory enables a rapid, sensitive, precise detection of CRC by the selective detection of miR-21 and miR-141, two important cancer markers and nano-molecules for cancer progression. Additionally, the non-invasiveness and low cost of the current sensor will benefit its wide application to clinical patients’ follow-ups or population screenings. Indeed, in our study, the small sample size limited higher-stage observation and analysis (only one case). Recognizing the importance and the minor pitfalls of this study, it is our opinion to emphasize that a large population study is necessary before this sensor can be applied in clinical diagnosis. Moreover, further studies and improvements to parameters are required in order to provide this technology to the frontlines against cancer.

## Data Availability

The data that support the findings of this study are available from the corresponding author upon reasonable request.

## References

[1] The Global Cancer Observatory (2020). All Cancers Excl. Non-Melanoma Skin Cancer.

[2] Allison J.E., Fraser C.G., Halloran S.P., Young G.P. (2014). Population screening for colorectal cancer means getting FIT: The past, present, and future of colorectal cancer screening using the fecal immunochemical test for hemoglobin (FIT). Gut Liver.

[3] Lee J.K., Liles E.G., Bent S., Levin T.R., Corley D.A. (2014). Accuracy of fecal immunochemical tests for colorectal cancer: Systematic review and meta-analysis. Ann. Intern. Med..

[4] Carroll M.R., Seaman H.E., Halloran S.P. (2014). Tests and investigations for colorectal cancer screening. Clin. Biochem..

[5] Kim D.H., Pickhardt P.J., Taylor A.J., Leung W.K., Winter T.C., Hinshaw J.L., Gopal D.V., Reichelderfer M., Hsu R.H., Pfau P.R. (2007). CT colonography versus colonoscopy for the detection of advanced neoplasia. N. Engl. J. Med..

[6] Waye J.D. (1992). Colonoscopy. CA A Cancer J. Clin..

[7] Dinges S.S., Hohm A., Vandergrift L.A., Nowak J., Habbel P., Kaltashov I.A., Cheng L.L. (2019). Cancer metabolomic markers in urine: Evidence, techniques and recommendations. Nat. Rev. Urol..

[8] Nemoto R., Kato T., Harada M., Shibata K., Kano M. (1982). Mass screening for urinary tract cancer with urine cytology. J. Cancer Res. Clin. Oncol..

[9] Morrissey J.J., Mellnick V.M., Luo J., Siegel M.J., Figenshau R.S., Bhayani S., Kharasch E.D. (2015). Evaluation of Urine Aquaporin-1 and Perilipin-2 Concentrations as Biomarkers to Screen for Renal Cell Carcinoma: A Prospective Cohort Study. JAMA Oncol..

[10] Lu T., Li J. (2017). Clinical applications of urinary cell-free DNA in cancer: Current insights and promising future. Am. J. Cancer Res..

[11] Pezzuto F., Buonaguro L., Buonaguro F.M., Tornesello M.L. (2018). The Role of Circulating Free DNA and MicroRNA in Non-Invasive Diagnosis of HBV- and HCV-Related Hepatocellular Carcinoma. Int. J. Mol. Sci..

[12] Mok Y., Matsushita K., Ballew S.H., Sang Y., Jung K.J., Lee S., Jee S.H., Coresh J. (2017). Kidney Function, Proteinuria, and Cancer Incidence: The Korean Heart Study. Am. J. Kidney Dis..

[13] Erbes T., Hirschfeld M., Rucker G., Jaeger M., Boas J., Iborra S., Mayer S., Gitsch G., Stickeler E. (2015). Feasibility of urinary microRNA detection in breast cancer patients and its potential as an innovative non-invasive biomarker. BMC Cancer.

[14] Gasparri M.L., Casorelli A., Bardhi E., Besharat A.R., Savone D., Ruscito I., Farooqi A.A., Papadia A., Mueller M.D., Ferretti E. (2017). Beyond circulating microRNA biomarkers: Urinary microRNAs in ovarian and breast cancer. Tumour Biol..

[15] Debernardi S., Massat N.J., Radon T.P., Sangaralingam A., Banissi A., Ennis D.P., Dowe T., Chelala C., Pereira S.P., Kocher H.M. (2015). Noninvasive urinary miRNA biomarkers for early detection of pancreatic adenocarcinoma. Am. J. Cancer Res..

[16] Falzone L., Gattuso G., Tsatsakis A., Spandidos D.A., Libra M. (2021). Current and innovative methods for the diagnosis of COVID-19 infection (Review). Int. J. Mol. Med..

[17] Yuan X., Sui G., Zhang D., Chen M., Zhao W. (2022). Recent developments and trends of automatic nucleic acid detection systems. J. Biosaf. Biosecur..

[18] Hakimian F., Ghourchian H., Hashemi A.S., Arastoo M.R., Behnam Rad M. (2018). Ultrasensitive optical biosensor for detection of miRNA-155 using positively charged Au nanoparticles. Sci. Rep..

[19] Pang S.N., Lin Y.L., Yu K.J., Chiou Y.E., Leung W.H., Weng W.H. (2021). An Effective SARS-CoV-2 Electrochemical Biosensor with Modifiable Dual Probes Using a Modified Screen-Printed Carbon Electrode. Micromachines.

[20] Palchetti I., Mascini M. (2008). Nucleic acid biosensors for environmental pollution monitoring. Analyst.

[21] Kerman K., Kobayashi M., Tamiya E. (2003). Recent trends in electrochemical DNA biosensor technology. Meas. Sci. Technol..

[22] Drummond T.G., Hill M.G., Barton J.K. (2003). Electrochemical DNA sensors. Nat. Biotechnol..

[23] Pray L. (2008). Discovery of DNA Structure and Function: Watson and Crick. Nat. Educ..

[24] Elbaz J., Tel-Vered R., Freeman R., Yildiz H.B., Willner I. (2009). Switchable motion of DNA on solid supports. Angew. Chem. Int. Ed. Engl..

[25] Santhanam M., Algov I., Alfonta L. (2020). DNA/RNA Electrochemical Biosensing Devices a Future Replacement of PCR Methods for a Fast Epidemic Containment. Sensors.

[26] He L., Hannon G.J. (2004). MicroRNAs: Small RNAs with a big role in gene regulation. Nat. Rev. Genet..

[27] Shen J., Wang A., Wang Q., Gurvich I., Siegel A.B., Remotti H., Santella R.M. (2013). Exploration of genome-wide circulating microRNA in hepatocellular carcinoma: MiR-483-5p as a potential biomarker. Cancer Epidemiol. Biomark. Prev..

[28] Chen X., Ba Y., Ma L., Cai X., Yin Y., Wang K., Guo J., Zhang Y., Chen J., Guo X. (2008). Characterization of microRNAs in serum: A novel class of biomarkers for diagnosis of cancer and other diseases. Cell Res..

[29] Lee Y.S., Dutta A. (2009). MicroRNAs in cancer. Annu. Rev. Pathol..

[30] Cheng H., Zhang L., Cogdell D.E., Zheng H., Schetter A.J., Nykter M., Harris C.C., Chen K., Hamilton S.R., Zhang W. (2011). Circulating plasma MiR-141 is a novel biomarker for metastatic colon cancer and predicts poor prognosis. PLoS ONE.

[31] O’Brien J., Hayder H., Zayed Y., Peng C. (2018). Overview of MicroRNA Biogenesis, Mechanisms of Actions, and Circulation. Front. Endocrinol..

[32] Wieckowski E., Whiteside T.L. (2006). Human tumor-derived vs dendritic cell-derived exosomes have distinct biologic roles and molecular profiles. Immunol Res..

[33] Hunter M.P., Ismail N., Zhang X., Aguda B.D., Lee E.J., Yu L., Xiao T., Schafer J., Lee M.L., Schmittgen T.D. (2008). Detection of microRNA expression in human peripheral blood microvesicles. PLoS ONE.

[34] Sohel M.H. (2016). Extracellular/Circulating MicroRNAs: Release Mechanisms, Functions and Challenges. Achiev. Life Sci..

[35] Li J.Y., Yong T.Y., Michael M.Z., Gleadle J.M. (2010). Review: The role of microRNAs in kidney disease. Nephrology.

[36] Lodes M.J., Caraballo M., Suciu D., Munro S., Kumar A., Anderson B. (2009). Detection of cancer with serum miRNAs on an oligonucleotide microarray. PLoS ONE.

[37] Catuogno S., Esposito C.L., Quintavalle C., Cerchia L., Condorelli G., De Franciscis V. (2011). Recent Advance in Biosensors for microRNAs Detection in Cancer. Cancers.

[38] Calore F., Fabbri M. (2012). MicroRNAs and cancer. Atlas Genet. Cytogenet. Oncol. Haematol..

[39] Bahnassy A.A., El-Sayed M., Ali N.M., Khorshid O., Hussein M.M., Yousef H.F., Mohanad M.A., Zekri A.-R.N., Salem S.E. (2017). Aberrant expression of miRNAs predicts recurrence and survival in stage-II colorectal cancer patients from Egypt. Appl. Cancer Res..

[40] Jiang J., Zheng X., Xu X., Zhou Q., Yan H., Zhang X., Lu B., Wu C., Ju J. (2011). Prognostic significance of miR-181b and miR-21 in gastric cancer patients treated with S-1/Oxaliplatin or Doxifluridine/Oxaliplatin. PLoS ONE.

[41] Zheng Q., Peskoe S.B., Ribas J., Rafiqi F., Kudrolli T., Meeker A.K., De Marzo A.M., Platz E.A., Lupold S.E. (2014). Investigation of miR-21, miR-141, and miR-221 expression levels in prostate adenocarcinoma for associated risk of recurrence after radical prostatectomy. Prostate.

[42] Dong G., Liang X., Wang D., Gao H., Wang L., Wang L., Liu J., Du Z. (2014). High expression of miR-21 in triple-negative breast cancers was correlated with a poor prognosis and promoted tumor cell in vitro proliferation. Med. Oncol..

[43] Mahmoud E.H., Fawzy A., RA A.E. (2018). Serum MicroRNA-21 Negatively Relates to Expression of Programmed Cell Death-4 in Patients with Epithelial Ovarian Cancer. Asian Pac. J. Cancer Prev..

[44] Taha M., Mitwally N., Soliman A.S., Yousef E. (2020). Potential Diagnostic and Prognostic Utility of miR-141, miR-181b1, and miR-23b in Breast Cancer. Int. J. Mol. Sci..

[45] Li W., Dong Y., Wang K.J., Deng Z., Zhang W., Shen H.F. (2020). Plasma exosomal miR-125a-5p and miR-141-5p as non-invasive biomarkers for prostate cancer. Neoplasma.

[46] Ardila H.J., Sanabria-Salas M.C., Meneses X., Rios R., Huertas-Salgado A., Serrano M.L. (2019). Circulating miR-141-3p, miR-143-3p and miR-200c-3p are differentially expressed in colorectal cancer and advanced adenomas. Mol. Clin. Oncol..

[47] Sun L.H., Tian D., Yang Z.C., Li J.L. (2020). Exosomal miR-21 promotes proliferation, invasion and therapy resistance of colon adenocarcinoma cells through its target PDCD4. Sci. Rep..

[48] Yin J., Bai Z., Song J., Yang Y., Wang J., Han W., Zhang J., Meng H., Ma X., Yang Y. (2014). Differential expression of serum miR-126, miR-141 and miR-21 as novel biomarkers for early detection of liver metastasis in colorectal cancer. Chin. J. Cancer Res..

[49] Ogata-Kawata H., Izumiya M., Kurioka D., Honma Y., Yamada Y., Furuta K., Gunji T., Ohta H., Okamoto H., Sonoda H. (2014). Circulating exosomal microRNAs as biomarkers of colon cancer. PLoS ONE.

[50] Feng Y.H., Tsao C.J. (2016). Emerging role of microRNA-21 in cancer. Biomed. Rep..

[51] de Klerk C.M., Vendrig L.M., Bossuyt P.M., Dekker E. (2018). Participant-Related Risk Factors for False-Positive and False-Negative Fecal Immunochemical Tests in Colorectal Cancer Screening: Systematic Review and Meta-Analysis. Am. J. Gastroenterol..

[52] van der Meulen M.P., Lansdorp-Vogelaar I., van Heijningen E.M., Kuipers E.J., van Ballegooijen M. (2016). Nonbleeding adenomas: Evidence of systematic false-negative fecal immunochemical test results and their implications for screening effectiveness-A modeling study. Cancer.

